# Directional prefrontal-thalamic information flow is selectively required during spatial working memory retrieval

**DOI:** 10.3389/fnins.2022.1055986

**Published:** 2022-11-23

**Authors:** Jia Wang, Shengnan Zhang, Tiaotiao Liu, Xuyuan Zheng, Xin Tian, Wenwen Bai

**Affiliations:** School of Biomedical Engineering and Technology, Tianjin Medical University, Tianjin, China

**Keywords:** spatial working memory, medial prefrontal cortex (mPFC), mediodorsal thalamus (MD), local field potentials (LFPs), information flow

## Abstract

**Introduction:**

Spatial working memory is a kind of short-term memory that allows temporarily storing and manipulating spatial information. Evidence suggests that spatial working memory is processed through three distinctive phases: Encoding, maintenance, and retrieval. Though the medial prefrontal cortex (mPFC) and mediodorsal thalamus (MD) are involved in memory retrieval, how the functional interactions and information transfer between mPFC and MD remains largely unclear.

**Methods:**

We recorded local field potentials (LFPs) from mPFC and MD while mice performed a spatial working memory task in T-maze. The temporal dynamics of functional interactions and bidirectional information flow between mPFC and MD was quantitatively assessed by using directed transfer function.

**Results:**

Our results showed a significantly elevated information flow from mPFC to MD, varied in time and frequency (theta in particular), accompanying successful memory retrieval.

**Discussion:**

Elevated theta information flow, a feature that was absent on error trials, indicates an important role of the directional information transfer from mPFC to MD for memory retrieval.

## Introduction

Working memory is defined as a brain system that provides temporary storage and manipulation of the information necessary for complex cognitive tasks (Baddeley, 1992, 2010; D’Esposito and Postle, 2015). Spatial working memory, especially, refers to the temporary online maintenance and/or manipulation of spatial information to be used toward a specific goal (van Asselen et al., 2006; Vance and Winther, 2021).

Cognitive neuroscientists have employed empirical and theoretical approaches to study the components of spatial working memory in attempts to localize and characterize the neural implementation. The process of spatial working memory is recognized typically divided into three phases: Encoding, maintenance, and retrieval (Spellman et al., 2015; Proskovec et al., 2016; Bolkan et al., 2017; Vogel et al., 2022). Specially, encoding involves the loading of information into working memory, while maintenance encompasses the active rehearsal of that information for a brief period of time. Finally, retrieval refers to the recall and application of the information to achieve a cognitive goal, which is also bound up with the updating of memory information in working memory and evaluation or monitoring of this information.

Extensive evidence has demonstrated the medial prefrontal cortex (mPFC) and mediodorsal thalamus (MD) is involved in working memory retrieval (Alexinsky, 2001; Horst and Laubach, 2009; Bolkan et al., 2017; Kupferschmidt and Gordon, 2018; Stout and Griffin, 2020). The reciprocal MD-mPFC activity is recognized to be crucial for successful execution of spatial working memory task (Parnaudeau et al., 2013, 2018; Jayachandran et al., 2019). Though anatomical evidence has shown the direct projections between MD and ventral-medial PFC (Mitchell, 2015; Bolkan et al., 2017), the question of how the information is parsed and dynamically transferred between the two areas to support working memory remains largely unclear.

Recently, brain networks have attracted increasing attention due to the potential to better characterize brain dynamics in cognition (Park and Friston, 2013; Beaty et al., 2016; Gilson et al., 2020; Wang et al., 2020; Fogel et al., 2021). The methods with brain network analysis can be useful tools to estimate the directional functional interactions among brain regions and explore the mechanism for integration in the brain (Zhang and Glascher, 2020; Osada et al., 2021; Chen et al., 2022). So far, brain network analysis method has been used to explore the mechanism underlying working memory in human and revealed the altered connectivity in functional networks during working memory performance persists following conclusion of that performance (Gordon et al., 2014; Moraschi et al., 2020; Braun et al., 2021). Previous studies have also shown the enhanced directional information transfer from hippocampus to prefrontal cortex is required for successful execution of a delayed non-match-to-place (DNMTP) task in rat (Xia et al., 2019). Therefore, the network analysis approaches could offer crucial insight into the nature of functional interactions and information transfer in the mPFC-MD network correlates of spatial working memory performance.

In the present study, we recorded the LFPs from mPFC and MD of mice as they performed a spatial working memory task. Then the bidirectional information flow between the mPFC and MD were quantitatively evaluated during the task epochs. This study is expected to reveal the directional information transfer between mPFC and MD underlying spatial working memory retrieval.

## Materials and methods

All experimental procedures were conducted in accordance with the Guide for Care and Use of Laboratory Animals and approved by the Tianjin Medical University Animal Care and Use Committee (license number: TMUaMEC 2021060).

### Subjects

Male adult C57BL/6 mice (aged 10–12 weeks) were used for the current study and provided by the Experimental Animal Center of Tianjin Medical University (Tianjin, China). Mice were group-housed (4–5 per cage) under a 12-h light-dark cycle. Except when food-restricted for the purpose of behavioral training and testing, all mice were given *ad libitum* access to food and water.

### Delayed non-match-to-place task

**FIGURE 1:**
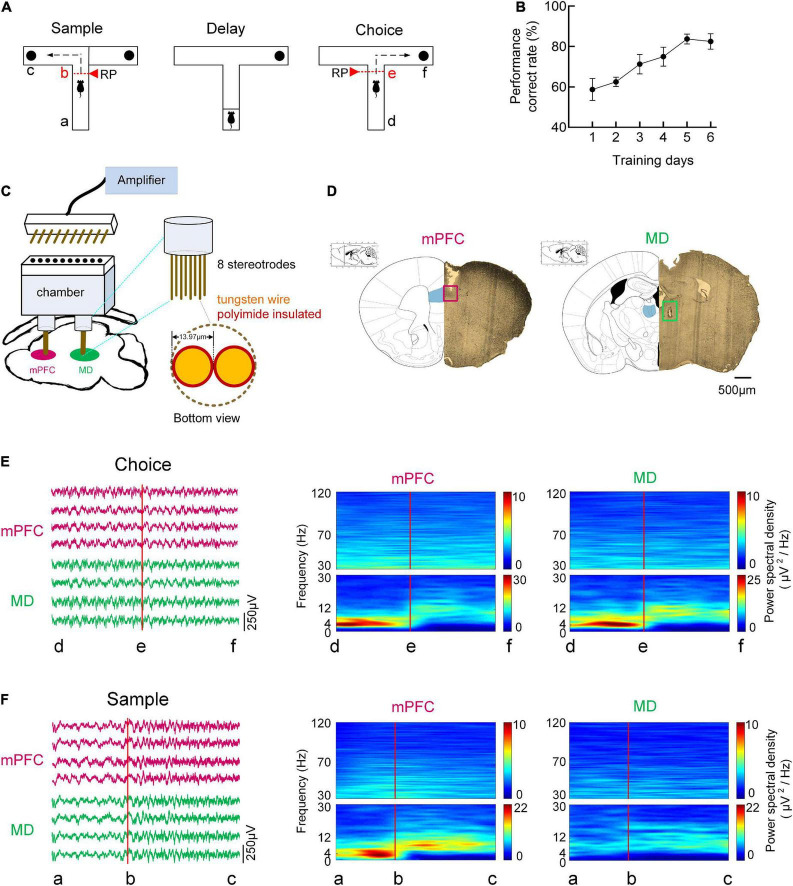
Behavioral task and oscillatory dynamics in mPFC and MD during the task. **(A)** Schema of a single trial of the DNMTP task in T-maze. In the sample/choice phase, the mouse’s initial position was defined as the point a/d, and the turn began was denoted as the point b/e, and the reward position was denoted as the point c/f. **(B)** Behavioral performance. The percentage of correct goal choice was enhanced as training progressed *n* = 8 mice. Error bars represent mean ± SEM. **(C)** Diagram for simultaneous multi-channel microelectrode recording. **(D)** Histological verification of the recording sites in the mPFC, PrL region (left) and MD (right). The partial brain sections show the typical recording sites (marked by rectangle) in the two regions. The superimposed schematics adapted from Paxinos and Charles (2005) show the coronal brain sections at 1.78 mm anterior and 1.22 mm posterior to the bregma. Scale bar, 500 μm. **(E)** Time-frequency power spectrum of LFPs during the choice phase on correct trials. (Left) Example single-trial LFP traces recorded from mPFC (purple) and MD (green). (Right) LFP power spectrum cross correct trials in mPFC and MD for a single mouse. **(F)** Same as **(E)**, but for sample phase.

Animals were trained on a DNMTP task in T-maze, a typical spatial working memory task for rodents, as described previously (Spellman et al., 2015; Bolkan et al., 2017). The schema of a single trial of the DNMTP task can be found in Figure 1A. A total of 3 days before the training, mice were gradually food restricted to maintain 85% of their body weight. Mice were then given 2 days of habituation to the maze, which consisted of 10 min free exploration and foraging with all doors open. On the subsequent 2 days mice underwent behavioral shaping consisting of 10 min of running to baited goal arms in alternating directions and back to the start box. Mice then underwent training on the T-maze task until criterion performance (consisting of 70% correct trials on 2 out of 3 consecutive days) was achieved (Spellman et al., 2015). In this experiment, intra-trial delay was 10 s and inter-trial delay was 20 s. The performances of the mice in the training sessions were measured by the accuracy of responses. The behavioral results showed that the mice (*n* = 8) learned the task gradually, improving from 58.8 ± 5.4% to 82.5 ± 3.9% correct choice over 6 days of training (Figure 1B).

### Electrodes array construction and implantation

Multi-electrode array for extracellular recording was constructed with polyimide insulated, tungsten wire (diameter: 13.97 μm, impedance <1 MΩ). All electrodes were referenced to a common electrode and common ground. For simultaneous recording of mPFC and MD, chamber was designed for accurate targeting to these two brain regions. For implantation, mice were deeply anesthetized with analgesics (Sodium pentobarbital, 10 mg/mL, 10 mg/kg i.p.) before surgery. Dura was carefully removed for the insertion of electrodes. According to the mouse brain atlas in stereotaxic coordinates, the stereotrodes were implanted in the mPFC (1.75 mm anterior, 0.4 mm lateral, and 1.8 mm ventral) and MD (1.2 mm posterior, 0.35 mm lateral, and 3.2 mm ventral). Finally, the electrodes were secured with dental cement. The recording sites of electrodes were further verified with histology after experiments.

### Neurophysiological recording

After electrodes implantation, mice were recovered for 1 week before neurophysiological recording. Recordings were made from mPFC and MD while the mice again performed the spatial working memory task (Figure 1C). Extracellular signals were recorded with the using the Cerebus Acquisition System (Blackrock Microsystems Inc., UT, USA), and the performance of the mice was obtained simultaneously by the overhead camera. For LFP recording, the signals were filtered with a pass-band of 0.5–120 Hz, further amplified and digitized at 1 kHz. Then polynomial fitting was used to remove the baseline drifts from LFPs and a 50-Hz notch filter was used to remove the powerline artifact. The behavioral data was also analyzed and the mouse’s initial position was defined as the point a/d, and the turn began was denoted as the point b/e, and the reward position was denoted as the point c/fduring sample/choice runs (marked in Figure 1A). For comparison, the neuronal activity in resting stage was also recorded and analyzed. In the present study, the resting stage was defined as the pre-task period in a rest box (Jadhav et al., 2016).

### Time-frequency spectral analysis

Spectral analysis was used to assess the dominant frequencies in the LFPs during the task. For averaged spectral density estimation during start-box, choice-point and reward occupancy, the multi-taper method was used (Jarvis and Mitra, 2001). For the assessment of power spectra, we used windows of 500 ms length that were moved over the data in steps of 125 ms, with 1 Hz resolution. Then the sub-bands were further obtained by band-pass filtering (Chebyshev filter).

### Functional connectivity in network and quantification of information flow

Directed transfer function (DTF) method, a multi-channel parametric method of analysis based on an autoregressive model, has been applied to a number of neurobiological systems for analysis of causal connectivity and flow pattern of activity in the frequency domain (Kaminski and Blinowska, 1991; Seth, 2010). In the present study, the electrodes were defined as the nodes of the causal network and elements of the DTF matrix were defined as the edges of the causal network, which were used to describe the network connectivity of the LFPs.

According to the multivariate autoregressive (MVAR) model, multi-channel LFPs can be represented as a data vector *X* of *N* source signals:


(1) $$X(t)={\left[x_{1}(t),x_{2}(t),\ldots ,x_{N}(t),\right]}^{T}$$

The MVAR model can then be expressed as (Bressler et al., 2021):


(2) $$X(t)=\sum_{n=1}^{p}A_{n}X(t-n)+E(t)$$

Where *p* is the model order, calculated by the Bayesian information criterion. *A_n_* is the coefficient matrix of the MVAR model. *E*(*t*) is the vector of multivariate zero mean uncorrelated white noise at time *t*.


(3) $$X(f)=A^{-1}(f)E(f)=H(f)E(f)$$

(4) $$H(f)=A^{-1}(f)=(\sum_{i=0}^{p}A(i)e^{-j2\pi fi\Delta t})$$

*f* denotes a specific frequency, *H*(*f*) is the transfer function matrix, and *A*(0) = −*I*, *I* is an identity matrix.


(5) $$\gamma_{ij}{}^{2}(f)=\frac{{\left|H(f)\right|}^{2}}{\sum_{m=1}^{k}{\left|H_{im}(f)\right|}^{2}}$$

γ_*ij*_(*f*) represents the ratio between the effect from channel *j* to channel *i*and the combined effect from all other nodes to node *j*, *k* is the number of nodes, and *H* is the transfer matrix of the system.


(6) $$DTF=\frac{1}{N(N-1)}\sum_{i\neq j\in G}DTF_{ij}$$

*DTF*_*ij*_ refers to the connectivity strength from channel *i* to channel *j*. *N* refers to the number of channels. *G* refers to the set of channels in the mPFC-MD causal network. In order to measure the information transfer in the network, the bidirectional information flow (*IF*) between mPFC and MD was calculated, respectively:


(7) $$IF_{mPFC\to MD}=\frac{1}{N_{mPFC}\times N_{MD}}\sum_{i\in N_{MD}}\sum_{j\in N_{mPFC}}DTF_{ij}$$

(8) $$IF_{MD\to mPFC}=\frac{1}{N_{MD}\times N_{mPFC}}\sum_{i\in N_{MD}}\sum_{j\in N_{mPFC}}DTF_{ji}$$

*IF*_*mPFC*→*MD*_ refers to the *IF* from the mPFC to the MD, and *IF*_*MD*→*mPFC*_ indicates the *IF* from the MD to the mPFC. *N*_*mPFC*_ and *N_MD_* refer to the number of channels in the mPFC and MD, respectively.

### Histology

After the experiments, the mice were deeply anesthetized and transcardially perfused with PBS (phosphate buffered solution) followed by 4% PFA (paraformaldehyde). Brain sections were mounted on slides to visualize and photograph lesions. Recording sites of the mPFC and MD were verified histologically (Figure 1D) and shown overlaid on a representative drawing [taken from the atlas of Paxinos and Charles (2005)].

### Data analysis and statistics

We simultaneously recorded LFPs from the mPFC and MD of eight mice as they performed the DNMTP task. In total, we describe 412 trials (including 295 correct trials and 117 incorrect trials) in the present article. The distribution of trials per subject is shown in Table 1. Data in the text and figures are expressed as the mean ± SEM. Statistical differences were evaluated by using Mann–Whitney test, two-way ANOVA, one-way ANOVA, and Bonferroni’s test for *post-hoc* analyses. *P*-values are marked statistically significant as follows: *P < 0.05, **P < 0.01, and ***P < 0.001. n.s., not significant.

**TABLE 1:** Distribution of trials per subject.

Mouse	Trials	
	
	Correct	Incorrect
1	42	18
2	48	18
3	22	12
4	50	13
5	34	13
6	41	18
7	29	12
8	29	13
Sum	295	117

## Results

### Increased theta oscillatory activity in medial prefrontal cortex and mediodorsal thalamus during memory retrieval

We simultaneously recorded LFPs from the mPFC and MD of eight mice using custom-made electrode arrays as they performed the DNMTP task that requires thalamic-prefrontal interactions. The examples of LFP traces recorded from the mPFC and MD were exhibited in Figure 1. Spectral analysis was used to assess the dominant frequencies in the LFPs during the task epochs. Figures 1E,F showed the time-frequency power spectrum across correct trials for a single mouse. A strong and transient increase in theta (4–12 Hz) activity in both mPFC and MD was observed preceded an animal’s correct choice during the choice phase. During the sample runs, however, the increased theta power was only found in mPFC but not MD. The time-frequency power spectrum reveals the prominent activity in the theta frequency band during working memory task as well as the mPFC and MD are involved in memory retrieval.

### Increased information flow in theta frequency band from medial prefrontal cortex to mediodorsal thalamus for correct choice

**FIGURE 2:**
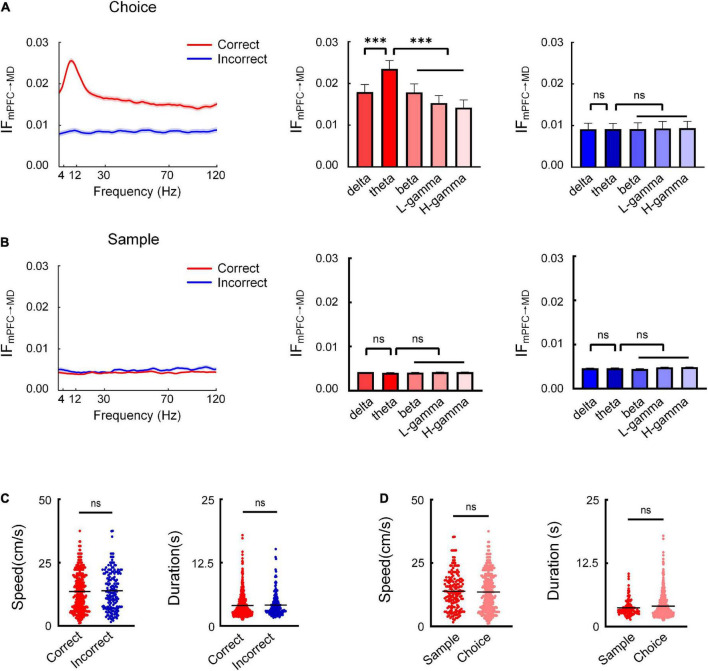
Theta-band information flow from mPFC to MD is prominent during the choice phase. **(A)** The average information flow across different frequencies during the choice phase. (Left) Information flow as a function of frequency across subjects (*n* = 8 mice). The red and blue curves represent the information flow on correct and incorrect trials, respectively. (Right) Comparison of information flow across different frequencies on correct and incorrect trials (correct: two-way AONVA, *F* = 55.02, *P* < 0.001; incorrect: two-way ANOVA, *P* > 0.05). **(B)** Same as **(A)**, but for the sample phase (correct: two-way AONVA, *P* > 0.05; incorrect: two-way ANOVA, *P* > 0.05). **(C)** Running speed and duration on correct and incorrect trials showed no significant difference (Mann–Whitney test, speed: *P* > 0.05; duration: *P* > 0.05). **(D)** Running speed and duration between the choice and sample phases showed no significant difference (Mann–Whitney test, speed: *P* > 0.05; duration: *P* > 0.05). ***P < 0.001, ns, not significant.

Information flows between mPFC and MD were assessed to reveal the nature of information transfer in the mPFC-MD network underlying spatial working memory. Firstly, the information flow across frequency bands from mPFC to MD for individual subject was reliably evaluated during the choice phase (Figure 2A and Supplementary Figure 1). To statistically quantify this result, we made a comparison of the average information flow between the correct and incorrect trials and the information flow in theta frequency band showed a the dominant role on correct trials (Figure 2A and Supplementary Tables 1,2). Moreover, the information flow in theta shows a remarkably increase on correct trials; however, no consistent difference among different frequency bands on incorrect trials across subjects (Figure 2A and Supplementary Figure 1).

To further investigate whether the enhanced theta-band information flow we observed is specific to the choice phase when memory retrieval is presumed to occur, we also analyzed the information flow during the sample phase. The results showed that there was no significant increase in the information flow between the mPFC and MD (Figure 2B). These findings suggested that the theta-band information transfer from mPFC to MD may serve the retrieval of working memory information.

In order to test whether the movements differ in the different trial types, we compared the running speed and the duration to choice between the correct and incorrect trials. The results showed no significant difference in the running speed and duration between the two the correct and incorrect trials (Figure 2C). The results indicated that the theta-band information flow were not influenced by animals’ locomotion we further compared the running speed between the choice run and sample sun. The results showed no significant difference in the running speed between the choice and sample phase (Figure 2D). These results suggest a role for theta-specific information transfer from mPFC to MD for successful memory retrieval.

We next subsampled the number of correct trials used for this analysis to match the number of incorrect trials for each animal and repeated this analysis to rule out potential differences to the number of trials in each condition. The increased theta-band information flow was also found in the re-sampled dataset. Moreover, the sub-sampled dataset of trials did not differ in running speed or duration (Supplementary Figure 4). The results showed that the difference in the numbers of correct and incorrect trials did not affect the analysis results.

### Dynamic changes in theta-specific information transfer from medial prefrontal cortex to mediodorsal thalamus during memory retrieval

**FIGURE 3:**
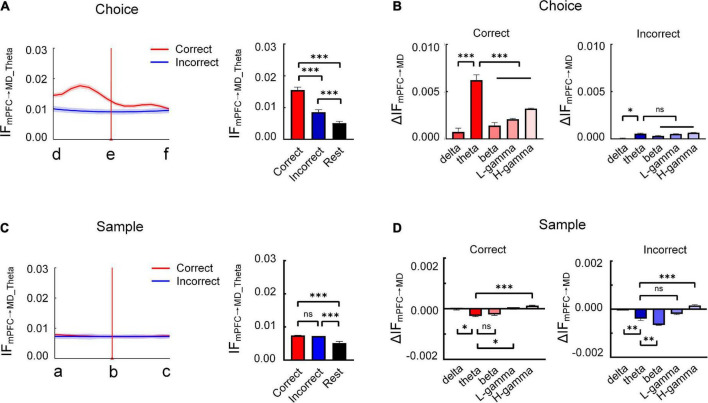
Enhanced theta-band information flow from mPFC to MD correlates with the task epochs. **(A)** The average theta-band information flow from mPFC to MD during the choice phase across subjects (*n* = 8 mice). (left) Changes in information flow over position [from start box (location “d”) to arrival at the reward port (location “f”)]. (Right) Comparison of theta-band information flow on correct, incorrect trials and rest stage (two-way ANOVA, *F* = 312.9, *P* < 0.001). **(B)** Theta-band information flow shows a remarkably increase on correct trials [ΔIF_mPFC→MD_ is defined as peak of IF (the maximum of IF during the choice period: d→e) minus the initial value of IF (at location “d”)]. Correct: two-way AONVA, *F* = 43.31, *P* < 0.001; incorrect: two-way ANOVA, *P* > 0.05. **(C)** same as **(A)**, but for sample phase. The information flow showed no difference in the different trial types and the values on correct and incorrect trials were apparently higher than that at rest stage (two-way ANOVA, F = 274.8, *P* < 0.001). **(D)** Same as **(B)**, but for sample phase [ΔIF_mPFC→MD_ is defined as peak of IF (the maximum of IF during the sample period: a→b) minus the initial value of IF (at location “a”)]. Correct: two-way AONVA, *F* = 12.13, *P* < 0.001; incorrect: two-way ANOVA, *F* = 44.29, *P* < 0.001. *P < 0.05, **P < 0.01, and ***P < 0.001. ns, not significant.

To gain more insights into how mPFC-MD information flow varies during memory retrieval, we next analyzed the temporal dynamics of information flow in theta band from mPFC to MD throughout choice phase. The information flow was found to experience the pattern of increase, peak, and decline on correct trials. Notably, the information flow was increased before the mice turned left or right during the choice phase on correct trials (Supplementary Figure 2A and Figure 3A). By contrast, no significant change in information flow was found on incorrect choices. We then compared the information flow in theta band between the trials with correct and incorrect choices. Averaged information flows on correct trials were statistically higher than those on incorrect trials. Besides, the measures of information flow during resting state were significantly lower than those in other conditions (Supplementary Figure 2B and Figure 3A, correct: 0.0155 ± 0.0009, incorrect: 0.0086 ± 0.0007, and rest: 0.0051 ± 0.0005, *P* < 0.001). These findings indicate that the temporal dynamics of theta-specific information transfer from mPFC to MD is required for successful memory retrieval. Moreover, the information flow in theta shows a remarkably increase during the choice phase on correct trials; however, no consistent difference among different frequency bands on incorrect trials across subjects (Figure 3B). For a better link the information flow we observed to a memory retrieval process, we further analyzed the information flow during the sample runs. The results showed that there was no significant increase in the information flow between the mPFC and MD (Figures 3C,D). These findings suggested that the theta-specific information transfer from mPFC to MD may serve the retrieval of working memory information.

### Directional medial prefrontal cortex-mediodorsal thalamus information flow is required during memory retrieval

**FIGURE 4:**
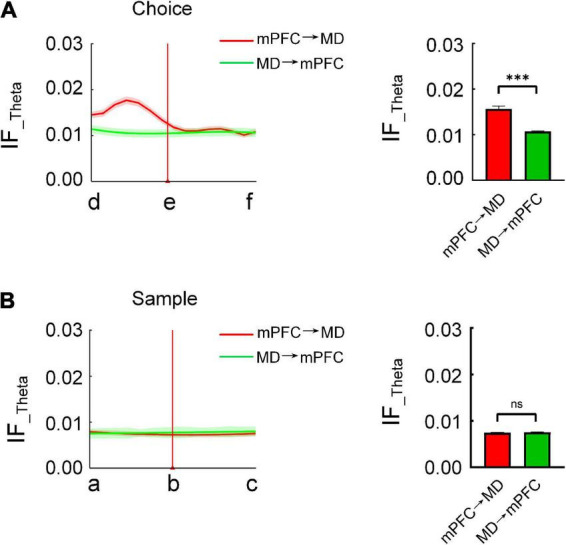
Directional information flow from mPFC to MD is distinct during the choice phase. **(A)** The bidirectional information flow between mPFC and MD across subjects (*n* = 8 mice) during the choice phase on correctly performed trials. (Left) The red and green curve represents the information flow from mPFC to MD and from MD to mPFC, respectively. (Right) The information flow from mPFC to MD was significantly higher than the reverse (Mann–Whitney test: *P* < 0.001). **(B)** Same as **(A)**, but for sample (Mann–Whitney test: *P* > 0.05). ***P < 0.001, ns, not significant.

In order to test the directionality of the coordinated information processing between the mPFC and MD, we further compared the bidirectional information flow (in theta frequency band) between mPFC and MD during the task epochs. The results showed that the information flow from mPFC to MD is differentially modulated during the choice phase. Specially, on correct trials, the information flow from mPFC to MD showed a markedly increase prior to the choice-points. By contrast, there was no distinct change in the reverse direction (Figure 4A and Supplementary Figure 3A). Statistical results showed the information flow from mPFC to MD was significantly higher than the reverse (Figure 4A and Supplementary Figure 3B, mPFC to MD: 0.0157 ± 0.0006, MD to mPFC: 0.011 ± 0.0001, Mann–Whitney test, *P* < 0.001). However, during the sample phase, there was no statistically significant change in the information flow between the mPFC and MD (Figure 4B). These findings demonstrate that the directional information transfer from mPFC to MD may serve the retrieval of working memory information.

## Discussion

In this study, we analyzed LFPs from mPFC and MD of mice performing a spatial working memory task and examined the temporal dynamics of functional interactions and directional information transfer between mPFC and MD during memory retrieval processing. Our results showed a significant increased information flow from mPFC to MD, varied in time and frequency, accompanying correct choice. Elevated theta information flow, a feature that was absent on error trials, indicates an important role of the directional information transfer from mPFC to MD for memory retrieval.

It is well known that successful spatial working memory performance depends on organized communication across expansive brain networks. The PFC, as a principal neural substrate of spatial working memory (Funahashi, 2017), has traditionally been proposed to function as a critical hub in these networks, wherein the information required for task is dynamically gated and integrated to guide activity in distal brain regions and support contextually tuned behavior (Miller and Cohen, 2001; Cole et al., 2012).

Emerging evidence indicates the thalamus has an extensive anatomical connectivity with the PFC. In particular, the MD (mediodorsal nucleus of the thalamus) shares dense, direct and reciprocal excitatory projections with various PFC subregions (Kupferschmidt and Gordon, 2018). In rodents, the medial segment of MD shares connections with the ventral-medial PFC (prelimbic and infralimbic cortices, medial orbitofrontal cortex). The central part of the MD is interconnected with the lateral-orbitofrontal cortex, and the lateral MD with the dorsal-medial PFC (anterior cingulate and accessory motor cortices) (Parnaudeau et al., 2018). This reciprocal connectivity is also selective as the MD receives its main cortical input from the PFC, and the PFC receives its main thalamic input from the MD (Mitchell, 2015). This bidirectional PFC-MD organization may facilitate information exchanges in spatial working memory tasks. Recent studies have revealed MD-PFC communication participates in working memory maintenance and retrieval (Miller et al., 2017; Kupferschmidt and Gordon, 2018). Especially, the mPFC-to-MD projections have been recognized to support memory retrieval or choice selection (Bolkan et al., 2017; Schmitt et al., 2017; Griffin, 2021).

Notably, hippocampus is another substrate which has robust long-range anatomical connectivity and functional interactions with the PFC (Hoover and Vertes, 2007; Bahner et al., 2015). Extensive evidences have demonstrated that hippocampal-prefrontal pathway is also crucial for spatial working memory. The anatomical studies showed the only direct projection between hippocampus and the mPFC is from the ventral hippocampus to the mPFC. Though there is no direct projection from the mPFC to the hippocampus, mPFC prominently projects to the Re, which sends projections to the hippocampus (Vertes, 2006; Vertes et al., 2006). Previous studies have found that direct monosynaptic projections from the ventral hippocampus to the mPFC support the encoding in working memory (Spellman et al., 2015).

Strikingly, in recent years, neuroscientists have made significant advances toward an understanding of the dynamic communication of the hippocampal-thalamo-PFC network that supports spatial working memory. The three different phases (encoding, maintenance, and retrieval) in working memory exhibit distinct functional interactions among brain networks. Specially, the PFC is generally involved in goal-directed actions, path planning, and strategy switching. The vHPC inputs to the mPFC support spatial encoding (Spellman et al., 2015), MD inputs to the mPFC support the maintenance of working memory by stabilizing task-relevant prefrontal activity during the delay period (Bolkan et al., 2017) and top-down signals from the mPFC back to the MD guide successful retrieve maintained information and translate it to motor action (Parnaudeau et al., 2018). Our present findings quantitatively revealed the increased information flow from mPFC and MD during correct choice, which is consistent with the theoretical frameworks that the mPFC-MD pathway supports the memory retrieval.

Another point worth discussing in this study is the theta-specific pattern for successful memory retrieval. Previous studies have revealed that theta oscillations play an essential role in working memory. In human, theta activity can be engaged by the working memory task (Herweg et al., 2020; Riddle et al., 2020; Ratcliffe et al., 2022). At many cortical locations, theta power rises sharply when working memory becomes required, is maintained throughout the memory task, and decreases when working memory is no longer required. Furthermore, theta power during encoding predicts subsequent recall (Sederberg et al., 2003). A recent study reported a significantly increased functional connectivity in theta frequency domains during complex audiovisual object encoding in a working memory task (Xie et al., 2021). In rodents, theta oscillations underlie a key physiological mechanism for mediating these coordinated interactions of different regions. Specially, theta oscillations can be modulated by spatial working memory and choice period–related increases in hippocampal-PFC theta synchrony have been reported in mice hippocampus (O’Neill et al., 2013). The phase-locking of mPFC neuronal firing to hippocampal theta oscillations and theta-phase coherence between mPFC and hippocampus were enhanced during correct choices (Sigurdsson et al., 2010; Hallock et al., 2016).

Recently, cross-frequency coupling (CFC) has been proposed as an index neural interactions of information gating and communication within and between broad networks in cognition (Siegel et al., 2012; Roux and Uhlhaas, 2014). Cross-frequency coupling between different frequency bands may constitute a flexible mechanism for combining information across different temporal scales within local cortical networks (Helfrich and Knight, 2016; Abubaker et al., 2021). Many studies have shown the cross-frequency interaction in different brain regions is required for working memory performance and theta-gamma coupling is the most commonly reported in this context (Alekseichuk et al., 2016; Tamura et al., 2017). In particular, theta-gamma phase-amplitude coupling (PAC), one of the most common representation of cross-frequency coupling, has been considered to be the potential mechanism underlying working memory (Fell and Axmacher, 2011; Farrokhi et al., 2022). Further research is needed to more thoroughly explore the complex interaction and cross-frequency information transfer in the hippocampal-thalamo-PFC network that supports spatial working memory.

## Data availability statement

The raw data supporting the conclusions of this article will be made available by the authors, without undue reservation.

## Ethics statement

The animal study was reviewed and approved by the Tianjin Medical University Animal Care and Use Committee (license number: TMUaMEC 2021060).

## Author contributions

XT, XZ, and WB designed the experiment. JW, SZ, and TL carried out the experiments. JW analyzed the data. JW and WB wrote the manuscript. All authors read and approved the final manuscript.

## Supplementary material

Supplementary Figure 1 Information flow from mPFC to MD varies with frequency during the choice phase. Each row represents the data for individual subject. Information flow in theta frequency band was the highest among all frequency bands on correct trials (one-way ANOVA, Mouse 1: *F* = 104.3, *P* < 0.001; Mouse 2: *F* = 242.4, *P* < 0.001; Mouse 3: *F* = 85.60, *P* < 0.001; Mouse 4: *F* = 52.19, *P* < 0.001; Mouse 5: *F* = 72.81, *P* < 0.001; Mouse 6: *F* = 31.55, *P* < 0.001; Mouse 7: *F* = 304.9, *P* < 0.001; and Mouse 8: *F* = 97.53, *P* < 0.001). No consistent difference was found among different frequency bands on incorrect trials (one-way ANOVA, Mouse 1: *F* = 60.19, *P* < 0.001; Mouse 2: *F* = 38.60, *P* < 0.001; Mouse 3: *F* = 2.515, *P* > 0.05; Mouse 4: *F* = 9.953, *P* < 0.001; Mouse 5: *F* = 9.089, *P* < 0.001; Mouse 6: *F* = 32.25, *P* < 0.001; Mouse 7: *F* = 76.41, *P* < 0.001; and Mouse 8: *F* = 366.1, *P* < 0.001).

Supplementary Figure 2 Theta-band information flow from mPFC to MD varies during the choice phase. **(A)** Information flow from mPFC to MD during the choice phase. The curves show the results from start box (location “d”) to arrival at the reward port (location “f”) and each panel represents individual subject. **(B)** Comparison of the information flow in the different conditions (One-way ANOVA, Mouse 1: *F* = 43.32, *P* < 0.001; Mouse 2: *F* = 96.72, *P* < 0.001; Mouse 3: *F* = 275.1, *P* < 0.001; Mouse 4: *F* = 149.9, *P* < 0.001; Mouse 5: *F* = 121.4, *P* < 0.001; Mouse 6: *F* = 271.6, *P* < 0.001; Mouse 7: *F* = 87.67, *P* < 0.001; and Mouse 8: *F* = 463.4, *P* < 0.001).

Supplementary Figure 3 Directional theta-band information flow from mPFC to MD is prominent during the choice phase. **(A)** Bidirectional information flow between mPFC and MD on correct trials. **(B)** Comparison of the information flow in the two directions (*t*-test, Mouse 1: *P* < 0.01; Mouse 2: *P* < 0.01; Mouse 3: *P* < 0.05; Mouse 4: *P* < 0.001; Mouse 5: *P* < 0.01; Mouse 6: *P* < 0.01; Mouse 7: *P* < 0.01; and Mouse 8: *P* < 0.001).

Supplementary Figure 4 Information flow from mPFC to MD for the subsampled dataset. **(A)** Information flow across different frequencies during the choice phase. (Left) Information flow as a function of frequency across subjects (*n* = 8 mice, 295 correct trials and 117 incorrect trials). (Right) Comparison of the information flow across different frequencies on correct and incorrect trials (correct: two-way AONVA, *F* = 55.02, *P* < 0.001; incorrect: two-way ANOVA, *P* > 0.05). **(B)** Same as **(A)**, but for subsampled dataset (*n* = 8 mice, 117 correct trials and 117 incorrect trials; correct: two-way AONVA, *F* = 69.87, *P* < 0.001; right, incorrect: two-way ANOVA, *P* > 0.05). **(C)** Comparison of running speed and duration during the choice phase (Mann–Whitney test: Speed *P* > 0.05; duration *P* > 0.05). **(D)** Same as **(C)**, but for subsampled dataset (Mann–Whitney test: Speed *P* > 0.05; duration: *P* > 0.05).
