# Supplementary material for: Directional prefrontal-thalamic information flow is selectively required during spatial working memory retrieval
Source: Front Neurosci. 2022 Nov 23;16:1055986. doi: 10.3389/fnins.2022.1055986 (PMC9726760; doi:10.3389/fnins.2022.1055986)
Supplement: Supplementary file 1 [file Table_1.doc]

| **Supplementary table1 Mean, standard deviation and coefficient of variation  for IF**mPFC→MD on the correct trials | | | | |
| --- | --- | --- | --- | --- |
| **Subject  No.** | **Frequency band** | **Mean** | **Standard deviation** | **Coefficient of variation** |
| **mouse 1** | **Delta** | **0.0179** | **0.0006** | **3.43%** |
|  | **Theta** | **0.0234** | **0.0019** | **7.93%** |
|  | **Beta** | **0.0174** | **0.0020** | **11.55%** |
|  | **Low-gamma** | **0.0155** | **0.0005** | **3.12%** |
|  | **High-gamma** | **0.0146** | **0.0009** | **6.04%** |
| **mouse 2** | **Delta** | **0.0145** | **0.0004** | **2.59%** |
|  | **Theta** | **0.0165** | **0.0006** | **3.74%** |
|  | **Beta** | **0.0117** | **0.0019** | **15.93%** |
|  | **Low-gamma** | **0.0080** | **0.0005** | **6.47%** |
|  | **High-gamma** | **0.0063** | **0.0004** | **5.85%** |
| **mouse 3** | **Delta** | **0.0120** | **0.0003** | **2.81%** |
|  | **Theta** | **0.0160** | **0.0016** | **9.69%** |
|  | **Beta** | **0.0105** | **0.0021** | **20.14%** |
|  | **Low-gamma** | **0.0086** | **0.0006** | **6.77%** |
|  | **High-gamma** | **0.0073** | **0.0008** | **10.25%** |
| **mouse 4** | **Delta** | **0.0248** | **0.0012** | **4.96%** |
|  | **Theta** | **0.0320** | **0.0019** | **5.94%** |
|  | **Beta** | **0.0261** | **0.0021** | **8.01%** |
|  | **Low-gamma** | **0.0224** | **0.0007** | **2.93%** |
|  | **High-gamma** | **0.0218** | **0.0009** | **4.07%** |
| **mouse 5** | **Delta** | **0.0255** | **0.0006** | **2.17%** |
|  | **Theta** | **0.0299** | **0.0014** | **4.82%** |
|  | **Beta** | **0.0252** | **0.0023** | **9.22%** |
|  | **Low-gamma** | **0.0195** | **0.0012** | **6.27%** |
|  | **High-gamma** | **0.0170** | **0.0005** | **2.73%** |
| **mouse 6** | **Delta** | **0.0162** | **0.0008** | **4.91%** |
|  | **Theta** | **0.0230** | **0.0023** | **10.09%** |
|  | **Beta** | **0.0167** | **0.0018** | **10.84%** |
|  | **Low-gamma** | **0.0158** | **0.0009** | **5.84%** |
|  | **High-gamma** | **0.0150** | **0.0010** | **6.81%** |
| **mouse 7** | **Delta** | **0.0192** | **0.0006** | **5.78%** |
|  | **Theta** | **0.0255** | **0.0006** | **6.57%** |
|  | **Beta** | **0.0202** | **0.0017** | **8.37%** |
|  | **Low-gamma** | **0.0183** | **0.0011** | **5.86%** |
|  | **High-gamma** | **0.0174** | **0.0009** | **5.32%** |
| **mouse 8** | **Delta** | **0.0136** | **0.0004** | **3.28%** |
|  | **Theta** | **0.0216** | **0.0031** | **14.50%** |
|  | **Beta** | **0.0154** | **0.0020** | **13.31%** |
|  | **Low-gamma** | **0.0144** | **0.0008** | **5.74%** |
|  | **High-gamma** | **0.0143** | **0.0011** | **7.40%** |
