# Supplementary material for: Directional prefrontal-thalamic information flow is selectively required during spatial working memory retrieval
Source: Front Neurosci. 2022 Nov 23;16:1055986. doi: 10.3389/fnins.2022.1055986 (PMC9726760; doi:10.3389/fnins.2022.1055986)
Supplement: Supplementary file 2 [file Table_2.doc]

| **Supplementary table 2 Mean, standard deviation and coefficient of variation  for IF**mPFC→MD on the incorrect trials | | | | |
| --- | --- | --- | --- | --- |
| **Subject  No.** | **Frequency band** | **Mean** | **Standard deviation** | **Coefficient of variation** |
| **mouse 1** | **Delta** | **0.0110** | **0.0003** | **3.11%** |
|  | **Theta** | **0.0115** | **0.0002** | **1.63%** |
|  | **Beta** | **0.0110** | **0.0004** | **3.21%** |
|  | **Low-gamma** | **0.0113** | **0.0010** | **9.20%** |
|  | **High-gamma** | **0.0114** | **0.0005** | **4.36%** |
| **mouse 2** | **Delta** | **0.0077** | **0.0001** | **1.50%** |
|  | **Theta** | **0.0078** | **0.0001** | **1.90%** |
|  | **Beta** | **0.0067** | **0.0004** | **6.08%** |
|  | **Low-gamma** | **0.0063** | **0.0003** | **5.02%** |
|  | **High-gamma** | **0.0069** | **0.0002** | **3.41%** |
| **mouse 3** | **Delta** | **0.0057** | **0.0000** | **0.22%** |
|  | **Theta** | **0.0055** | **0.0001** | **2.53%** |
|  | **Beta** | **0.0048** | **0.0003** | **5.51%** |
|  | **Low-gamma** | **0.0038** | **0.0003** | **7.48%** |
|  | **High-gamma** | **0.0036** | **0.0001** | **1.85%** |
| **mouse 4** | **Delta** | **0.0127** | **0.0001** | **0.44%** |
|  | **Theta** | **0.0123** | **0.0005** | **4.15%** |
|  | **Beta** | **0.0136** | **0.0014** | **10.46%** |
|  | **Low-gamma** | **0.0153** | **0.0004** | **2.91%** |
|  | **High-gamma** | **0.0156** | **0.0004** | **2.86%** |
| **mouse 5** | **Delta** | **0.0066** | **0.0000** | **0.51%** |
|  | **Theta** | **0.0066** | **0.0001** | **1.09%** |
|  | **Beta** | **0.0062** | **0.0001** | **1.38%** |
|  | **Low-gamma** | **0.0069** | **0.0004** | **5.21%** |
|  | **High-gamma** | **0.0066** | **0.0004** | **5.32%** |
| **mouse 6** | **Delta** | **0.0119** | **0.0001** | **0.61%** |
|  | **Theta** | **0.0123** | **0.0006** | **4.91%** |
|  | **Beta** | **0.0131** | **0.0004** | **3.12%** |
|  | **Low-gamma** | **0.0135** | **0.0011** | **8.23%** |
|  | **High-gamma** | **0.0133** | **0.0010** | **7.46%** |
| **mouse 7** | **Delta** | **0.0053** | **0.0001** | **2.24%** |
|  | **Theta** | **0.0055** | **0.0001** | **1.14%** |
|  | **Beta** | **0.0050** | **0.0002** | **4.51%** |
|  | **Low-gamma** | **0.0050** | **0.0004** | **8.78%** |
|  | **High-gamma** | **0.0049** | **0.0004** | **8.49%** |
| **mouse 8** | **Delta** | **0.0028** | **0.0000** | **0.59%** |
|  | **Theta** | **0.0034** | **0.0004** | **10.55%** |
|  | **Beta** | **0.0037** | **0.0003** | **8.11%** |
|  | **Low-gamma** | **0.0033** | **0.0003** | **7.82%** |
|  | **High-gamma** | **0.0040** | **0.0005** | **13.09%** |
