# Supplementary figures and images for: Directional prefrontal-thalamic information flow is selectively required during spatial working memory retrieval
Source: Front Neurosci. 2022 Nov 23;16:1055986. doi: 10.3389/fnins.2022.1055986 (PMC9726760; doi:10.3389/fnins.2022.1055986)

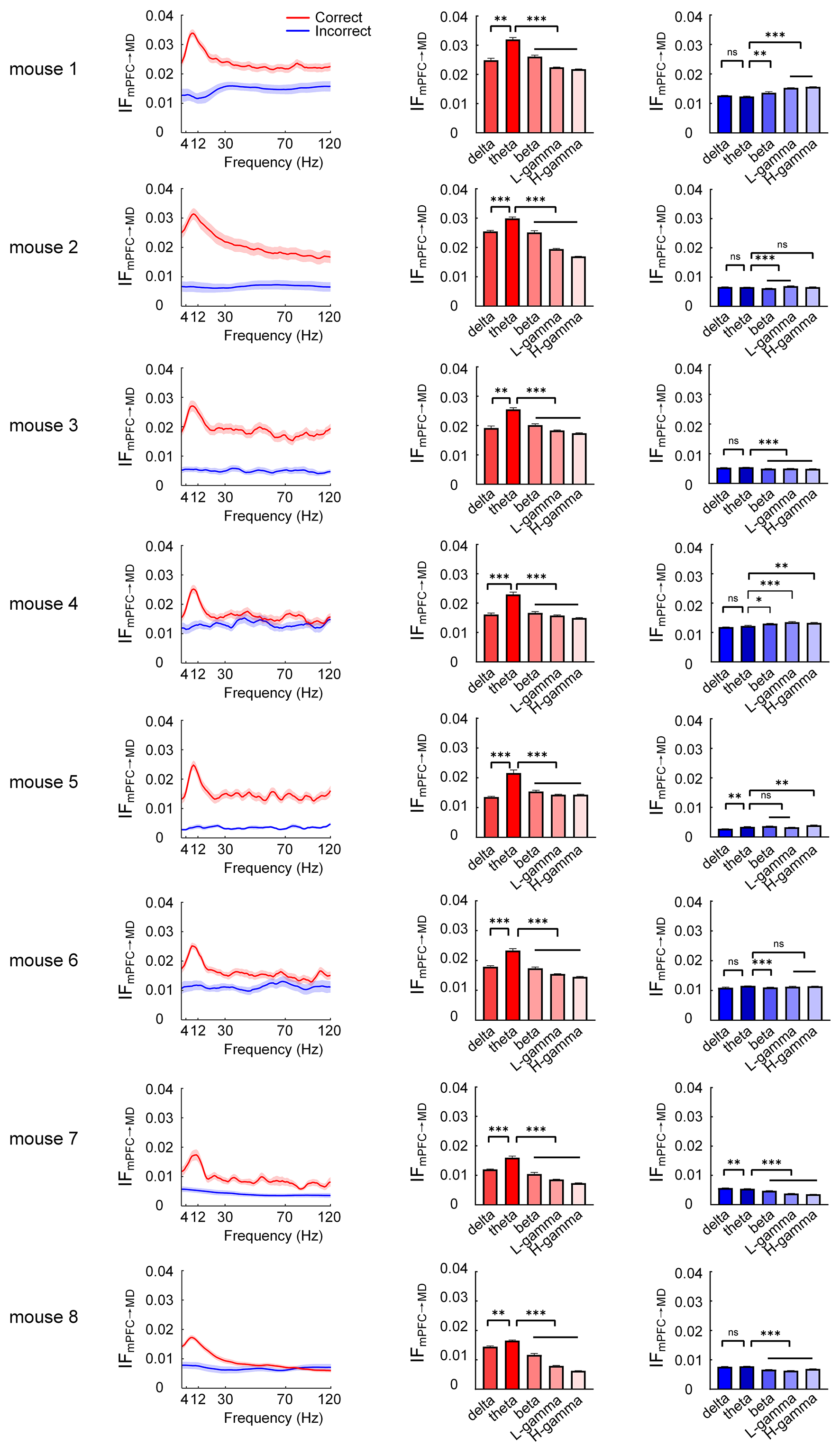

Supplement: Supplementary Figure 1 — Information flow from mPFC to MD varies with frequency during the choice phase. Each row represents the data for individual subject. Information flow in theta frequency band was the highest among all frequency bands on correct trials (one-way ANOVA, Mouse 1: F = 104.3, P < 0.001; Mouse 2: F = 242.4, P < 0.001; Mouse 3: F = 85.60, P < 0.001; Mouse 4: F = 52.19, P < 0.001; Mouse 5: F = 72.81, P < 0.001; Mouse 6: F = 31.55, P < 0.001; Mouse 7: F = 304.9, P < 0.001; and Mouse 8: F = 97.53, P < 0.001). No consistent difference was found among different frequency bands on incorrect trials (one-way ANOVA, Mouse 1: F = 60.19, P < 0.001; Mouse 2: F = 38.60, P < 0.001; Mouse 3: F = 2.515, P > 0.05; Mouse 4: F = 9.953, P < 0.001; Mouse 5: F = 9.089, P < 0.001; Mouse 6: F = 32.25, P < 0.001; Mouse 7: F = 76.41, P < 0.001; and Mouse 8: F = 366.1, P < 0.001). [file Image_1.TIF]

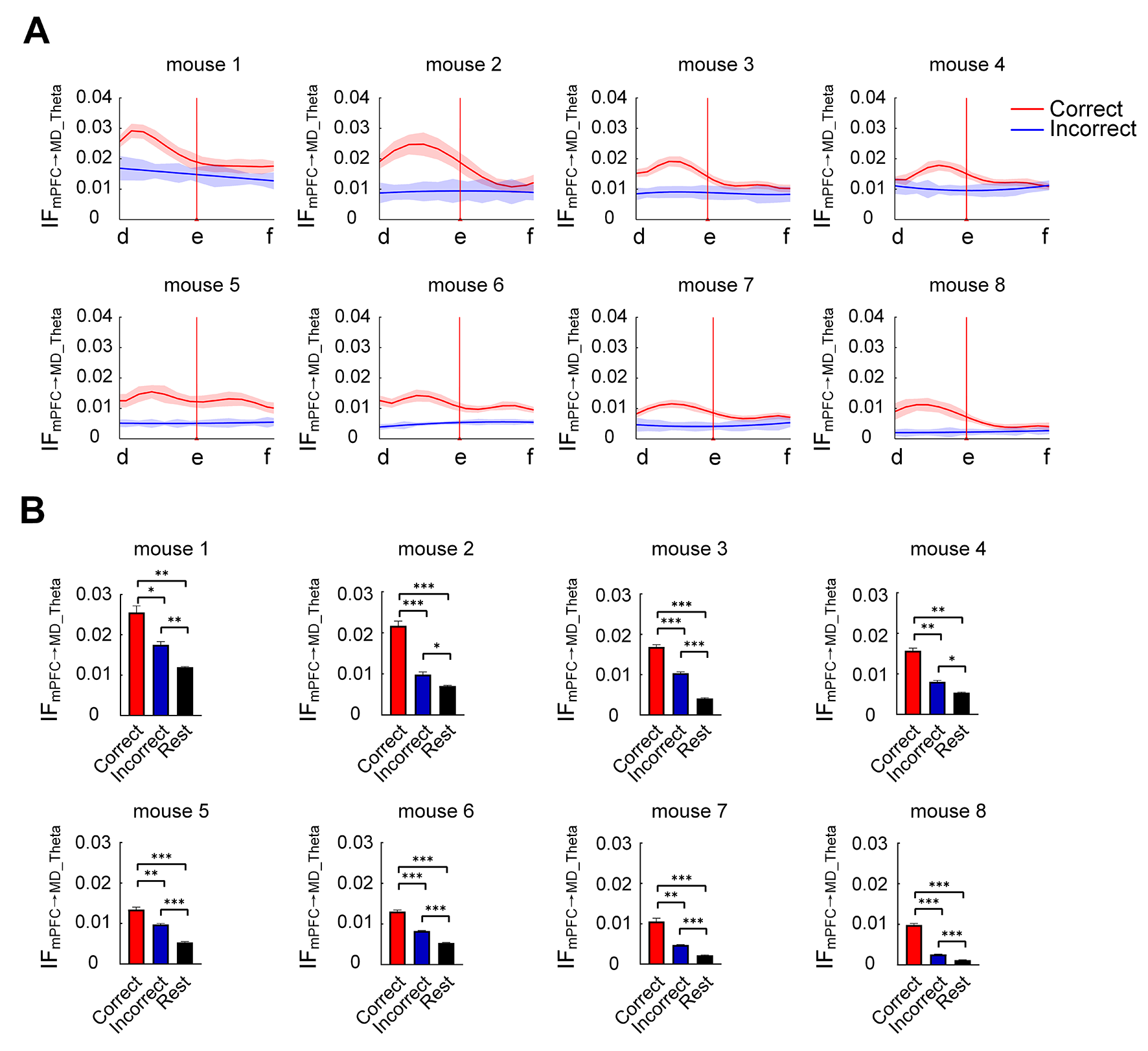

Supplement: Supplementary Figure 2 — Theta-band information flow from mPFC to MD varies during the choice phase. (A) Information flow from mPFC to MD during the choice phase. The curves show the results from start box (location “d”) to arrival at the reward port (location “f”) and each panel represents individual subject. (B) Comparison of the information flow in the different conditions (One-way ANOVA, Mouse 1: F = 43.32, P < 0.001; Mouse 2: F = 96.72, P < 0.001; Mouse 3: F = 275.1, P < 0.001; Mouse 4: F = 149.9, P < 0.001; Mouse 5: F = 121.4, P < 0.001; Mouse 6: F = 271.6, P < 0.001; Mouse 7: F = 87.67, P < 0.001; and Mouse 8: F = 463.4, P < 0.001). [file Image_2.TIF]

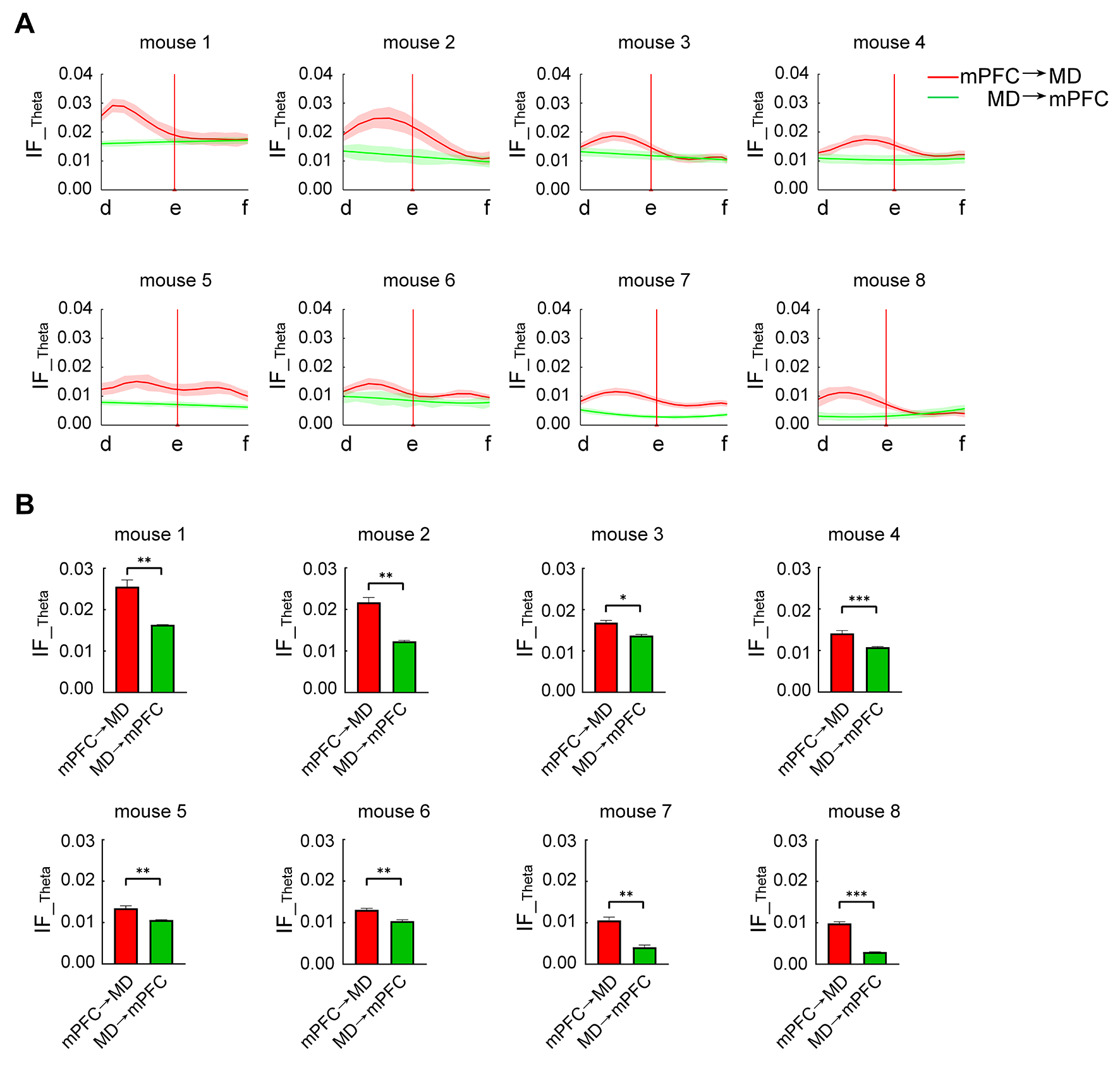

Supplement: Supplementary Figure 3 — Directional theta-band information flow from mPFC to MD is prominent during the choice phase. (A) Bidirectional information flow between mPFC and MD on correct trials. (B) Comparison of the information flow in the two directions (t-test, Mouse 1: P < 0.01; Mouse 2: P < 0.01; Mouse 3: P < 0.05; Mouse 4: P < 0.001; Mouse 5: P < 0.01; Mouse 6: P < 0.01; Mouse 7: P < 0.01; and Mouse 8: P < 0.001). [file Image_3.TIF]

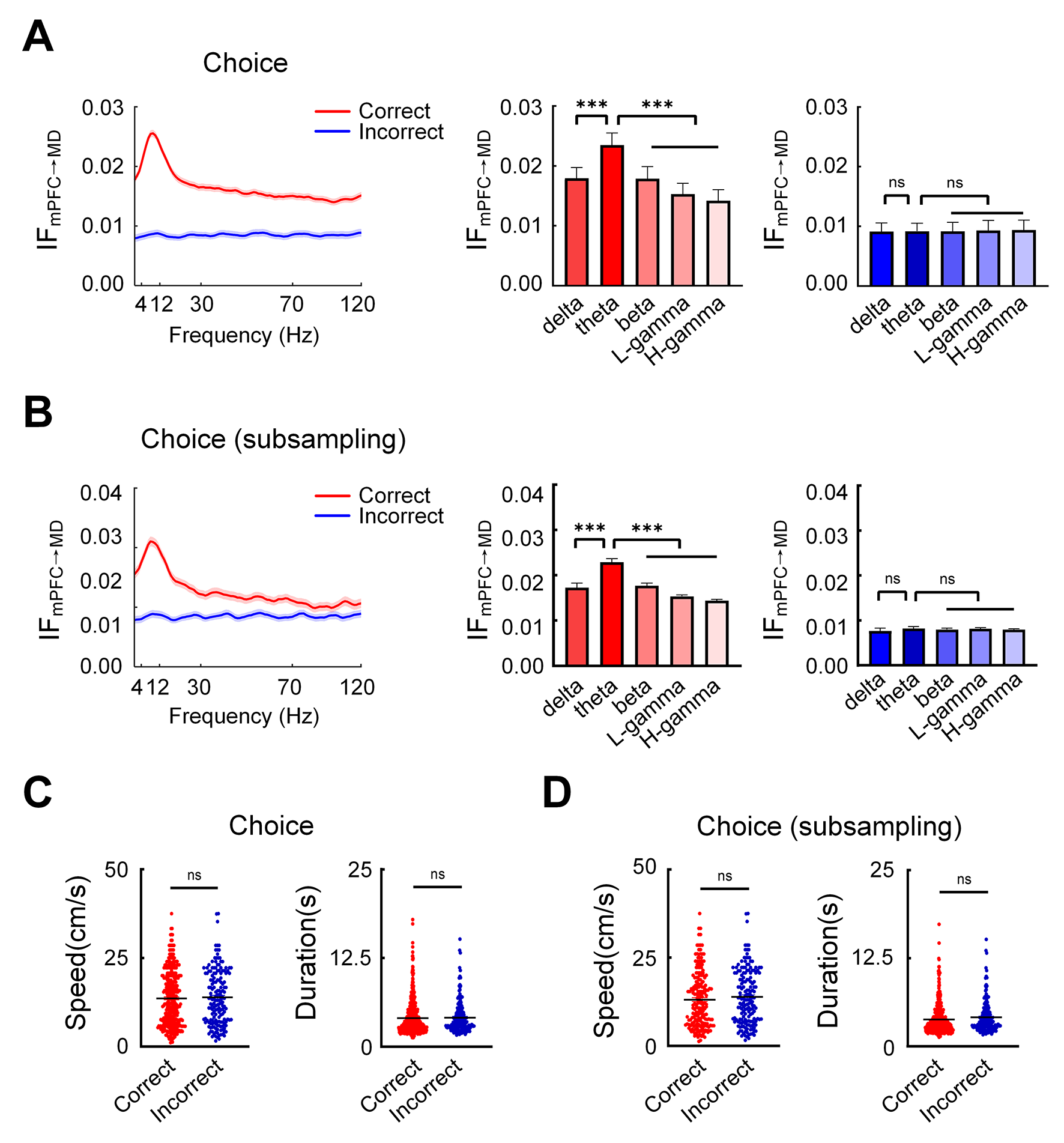

Supplement: Supplementary Figure 4 — Information flow from mPFC to MD for the subsampled dataset. (A) Information flow across different frequencies during the choice phase. (Left) Information flow as a function of frequency across subjects (n = 8 mice, 295 correct trials and 117 incorrect trials). (Right) Comparison of the information flow across different frequencies on correct and incorrect trials (correct: two-way AONVA, F = 55.02, P < 0.001; incorrect: two-way ANOVA, P > 0.05). (B) Same as (A), but for subsampled dataset (n = 8 mice, 117 correct trials and 117 incorrect trials; correct: two-way AONVA, F = 69.87, P < 0.001; right, incorrect: two-way ANOVA, P > 0.05). (C) Comparison of running speed and duration during the choice phase (Mann–Whitney test: Speed P > 0.05; duration P > 0.05). (D) Same as (C), but for subsampled dataset (Mann–Whitney test: Speed P > 0.05; duration: P > 0.05). [file Image_4.TIF]
